# An androgen reduced transcript of LncRNA GAS5 promoted prostate cancer proliferation

**DOI:** 10.1371/journal.pone.0182305

**Published:** 2017-08-03

**Authors:** Yingyi Zhang, Xinya Su, Zhe Kong, Fangqiu Fu, Pu Zhang, Dan Wang, Hai Wu, Xuechao Wan, Yao Li

**Affiliations:** 1 Department of Oncology, Changhai Hospital, Second Military Medical University, 168 Changhai Road, Shanghai, People’s Republic of China; 2 State Key Laboratory of Genetic Engineering, Institute of Genetics, School of Life Sciences, Fudan University, Shanghai, People’s Republic of China; Northern Institute for Cancer Research, UNITED KINGDOM

## Abstract

Prostate cancer (PCa) becomes a leading cause of death in males nowadays. Recent reports showed that androgen-responsive long non-coding RNAs played important roles in tumorigenesis and progression of PCa. In this study, we focused on a special transcript of GAS5 (ENST00000456293.5, GAS5-007), which was reported as a tumor suppressor. Here, we demonstrated GAS5-007 was reduced by androgen treatment and inhibited by AR. Next, we explored the expression level of GAS, finding the expression of it in PCa tissue was higher than normal tissue in both public databases and human tissue samples. Functional analysis of GAS5 showed it was related to regulating translational elongation, protein biosynthesis, and transcription. Moreover, we observed GAS5-007 knockdown inhibited the proliferation, cell cycle and promoted cell apoptosis of PCa. We also constructed a GAS5-miRNA network to explain the different roles of different GAS5 transcripts in PCa. This study provides novel insights to identify potential diagnostic biomarker and therapy target for prostate cancer in clinical treatment.

## Introduction

Prostate cancer (PCa) is a kind of most common epithelial malignancies in male worldwide. According to Chen et al.’s report, PCa was the seventh in incidence and the tenth in mortality in malignant tumors in males in China[1]. Substantial evidences had showed androgen receptor (AR) and its signaling pathway played pivotal roles in development and progression of prostate cancer[2]. However, the accurate molecular mechanisms of PCa progression remain unclear.

Long non-coding RNA (LncRNA) is a class of non-coding RNA with more than 200 nucleotides and involved in the progression of different kinds of cancer[3–7]. As for prostate cancer, for example, LncRNA PCAT5 has been described as an oncogene in ERG-positive PCa with implications for biomarker[8]. Recently, plenty of LncRNAs, including PCGEM1[9], SOCS2-AS1[10], PlncRNA-1[11], CTBP1-AS[12], DRAIC and PCAT29[13], have been reported to be associated with AR pathway, and thus acted as regulators in the development of PCa. HOTAIR, an androgen-repressed LncRNA, has been demonstrated to prevent AR ubiquitination and degradation by blocking the interaction between AR and the E3 ubiquitin ligase MDM2[14]. In our previous reports, we also identified a series of androgen-responsive lncRNAs and found LINC01138 and SUZ12P1 promoted the proliferation of PCa[15]. Follow-up researches are still needed to elucidate molecular mechanisms of the process of PCa and the effects of LncRNAs in it.

The growth arrest-specific transcript 5 (GAS5) has been brought under the spotlight in recent studies, suggesting it may have pivotal roles in various kinds of tumor. According to previous researches, GAS5 has been reported as a tumor suppressor[16–19]. In prostate cancer, there have been studies demonstrated that two transcripts of GAS5 (GAS5-O1 and GAS5-AE) promoted the apoptosis of prostate cancer cells[20]. In the latest report, GAS5 decelerated PCa development by targeting miR-103 through AKT/mTOR signaling pathway[21]. However, there is still lack of exact mechanisms about how lncRNA GAS5 experts its functions. Also, there is no sufficient research about the molecular functions of different transcripts of GAS5, which may play unique roles in cancers.

In our research, we focused on studying a particular transcript of GAS5 (ENST00000456293.5, GAS5-007) according to our previous finding and TCGA database. Initially, we examined the regulation of AR on this transcript of GAS5, finding out that the expression of it was reduced by AR. Next, to probe functions of it, we used both prediction through GO, KEGG pathway analysis and functional experiment, including CCK-8 assay, cell cycle and apoptosis analysis. We hope that our study will provide a potential new therapeutic and prognostic target for prostate cancer.

## Material and method

### Patients and tissue samples

The trial was approved by the Research Ethics Committee of Tongji Hospital and written consent was acquired from all tested patients. Eleven normal tissue and fourteen tumor tissues were used for qRT-PCR. All of the samples were collected from Tongji Hospital, a subsidiary of Shanghai Tongji University, between January 2001 and December 2013. These patients underwent radical prostatectomy but did not receive any pre-operation treatment. The histopathological features of tumor specimens were classified according to the Gleason score system and 2002 TNM classification system.

### Cell lines and cell culture

Human prostate cancer cell lines LNCaP, 22RV1, DU145, PC3 and normal prostate epithelial cell WPMY-1 were used in this study. LNCaP cells were purchased from the American Type Culture Collection (Manassas, USA) which was confirmed by short tandem repeat (STR) analysis. 22RV1, DU145, PC-3 and WPMY-1 were obtained from Cell Bank of Chinese Academy of Sciences (Shanghai, China) where they were authenticated by mycoplasma detection, DNA-Fingerprinting, isozyme detection and cell vitality detection. The four prostate cancer cells were cultured in RPMI-1640 medium (Corning, USA) with 10% FBS (Hyclone, USA) and WPMY-1 in DMEM medium (Corning, USA) with 10% FBS in a humidified atmosphere containing 5% CO2 at 37°C.

### RNA interference and transient transfection

All siRNA against AR, a siRNA targeting GAS5 and a siRNA negative control (NC) were purchased from GenePharma (Shanghai, China) and used at 50 nM concentration. Transfection was carried out using Lipofectamine 2000 Transfection Reagent (Life, USA), following the instruction of manufacturer. After 48h, knocking down of AR or GAS5 was confirmed by qRT-PCR. The following siRNAs were used: siGAS5: 5′- GCAGACCUGUUAUCCUAAATT-3′ and 5′- UUUAGGAUAACAGGUCUGCTT-3′; and a scrambled siRNA control: 5′-UUCUCCGAACGUGUCACGUTT-3′ and 5′-ACGUGACACGUUCGGAGAATT-3′.

### Real-time quantitative reverse transcription PCR (qRT-PCR) analysis

Total RNAs were extracted by using TRIzol reagent (Sigma). Reverse transcript PCR was performed by NovoScript® 1st Strand cDNA Synthesis SuperMix (Novoprotein Scientific Inc.,China). Real-time quantitative PCR was carried out through AceQ qPCR SYBR Green Master Mix (Vazyme Biotech co.,ltd) on Roche LightCycler 480. Primers for GAS5 were: forward, 5′-ACTCAAGCCATTGGCACAC-3′ and reverse, 5′-GCCACAGATGAGTGTTCACC-3′. Primers for β-actin were: forward, 5′-CCTCTCCCAAGTCCACACAG-3′ and reverse, 5′-GGGCACGAAGGCTCATCATT-3′. Primers were synthesized by SangonSBS Gentech Biotech (Shanghai, China). The Ct values were normalized to estimate different expression level of genes with β-actin as internal control. Relative mRNA expression level was calculated by the 2^−ΔΔ*Ct*^ method. To ensure the quantitative accuracy, each sample was run in triplicate.

### Cell proliferation assay

Cell proliferation analysis was performed with Cell Counting Kit-8 (CCK-8, Dojindo Laboratories, Kumamoto, Japan) in octuplicate according to the instructions of manufacturer. LNCaP cells of 5000 per well were seeded into 96-well plate, and examined at the time point of 0, 24, 48, 72h. At each time point, CCK-8 (10 μl) was added to the wells, and after an incubation of 2h at 37°C, absorbance was measured at 450nm with a Microplate Reader ELx808 (Bio-Tek, VT, USA).

### Cell cycle and apoptosis analysis

LNCaP and PC-3 cells were plated in 6-well plates in antibiotic-free medium and transfected with siRNA targeting GAS5 (siGAS5) or a negative control (NC) with Lipofectamine 2000 (Invitrogen). After 48h’s culture, cells were harvested and washed with PBS for three times. In cell cycle assay, cells were incubated with 0.03% triton X-100 and propidium iodide (PI) (50 ng/mL) for 15 min; the percentages of cells in different phases of cell cycle were measured with a FACScalibur flow cytometer (BD, CA, USA) and analyzed with ModFit software (Verity Software House, ME, USA). As for apoptosis assay, cells were assayed with FITC Annexin V Apoptosis Detection Kit (BD, CA, USA) and analyzed by flow cytometry. All experiments were conducted in triplicate.

### Statistical analysis

The numerical data were presented as mean ± standard deviation (SD) of at least three determinations. Statistical comparisons between groups of normalized data were performed using T-test or Mann–Whitney U-test according to the test condition. A p < 0.05 was considered statistical significance with a 95% confidence level.

## Results

### AR inhibited the expression of LncRNA GAS5

**Fig 1:**
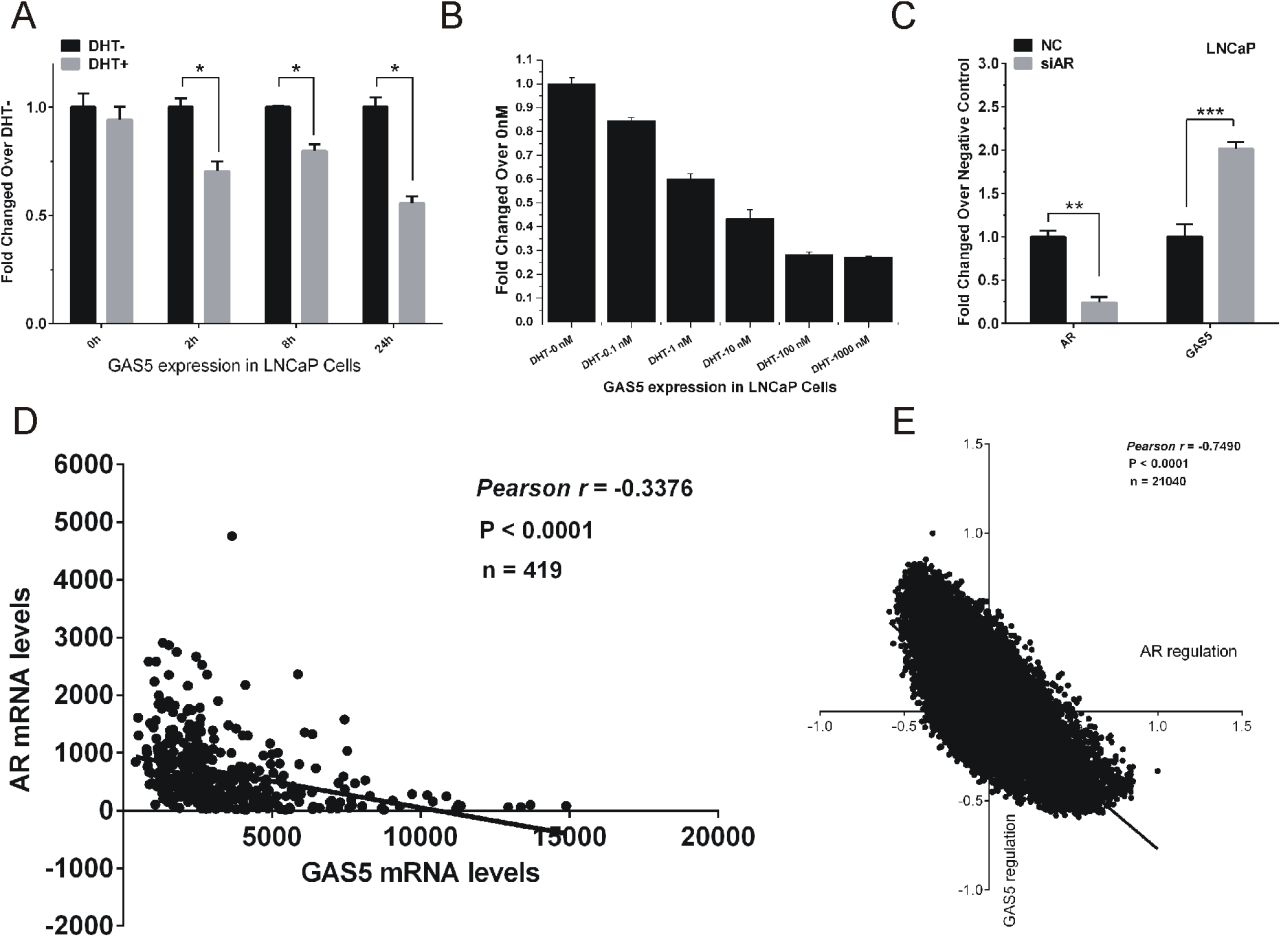
The regulation of AR on the expression of LncRNA GAS5. (A) qRT-PCR analysis of GAS5’s expression in LNCaP cells treated with DHT in time series of 0h, 2h, 8h, 24h, 48h. (B) qRT-PCR analysis of GAS5’s expression in LNCaP cells treated with DHT in dose series of 0nM, 0.1nM, 1nM, 10nM, 100nM, 1000nM. (C) The efficiency of siAR on the expression of AR was confirmed by qRT-PCR and the expression of GAS5 was tested after transfection of siAR compared with NC in LNCaP cells. (D) The correlation of mRNA level between GAS5 and AR. (E) The correlation of regulation between GAS5 and AR. Significance was defined as p < 0.05 (*p < 0.05; **p < 0.01; ***p < 0.001).

Dynamic expression levels of lncRNAs in LNCaP cells treated with dihydrotestosterone (DHT) have been reported previously. GAS5-007 was showed to be a dysregulated lncRNA in our previous study. To further confirm the early selection, we tested the expression of GAS5 under DHT treatment in LNCaP cells in a time series of 0h, 2h, 8h and 24h and found that the expression of GAS5-007 was downregulated after DHT stimulation (Fig 1A). We also found that the expression of GAS5-007 was gradually decreased with the gradient increase of DHT concentration from 0.1nM to 1000nM (Fig 1B), implying that it was regulated by AR. To further investigate the regulation of AR on GAS5-007, we performed siRNA-directed knockdown of AR in LNCaP cells and observed the expression of GAS5-007 was upregulated after knockdown of AR in LNCaP cells (Fig 1C).

To gather more evidence for the AR’s repression on GAS5, we took analysis of TCGA database. A significantly inverse relationship between AR and GAS5 was identified in PCa tissues (Fig 1D). In addition, we also found there was a negative correlation between GAS5 and AR regulation, suggesting that GAS5 may affect AR transcriptional activity (Fig 1E).

### LncRNA GAS5 was upregulated in prostate cancer tissue samples

**Fig 2:**
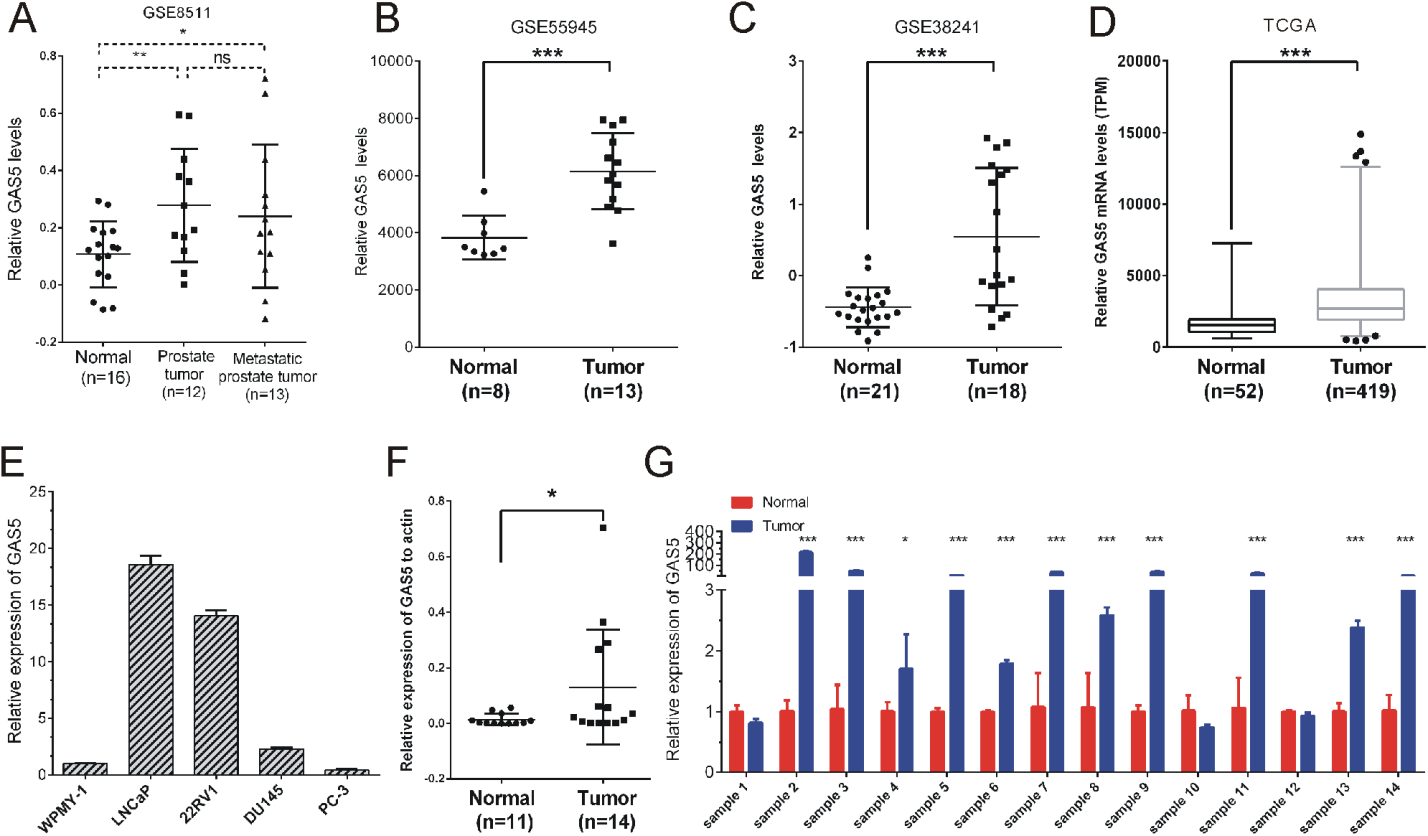
The expression of GAS5 in prostate cancer. (A) The relative expression level of GAS5 in tumor tissue compared with normal tissue in public gene expression data GSE8511, GSE55945 (B) and GSE38241 (C). (D) The relative mRNA level of GAS5 in 52 normal tissue samples compared with 419 tumor tissue samples from TCGA database. (E) Relative expression level of GAS5 was measured in normal prostate epithelial cell line WPMY-1 and four prostate cancer cell lines, LNCaP, 22RV1, DU145 and PC-3 by qRT-PCR. (F) The relative expression of GAS5 in 11 normal prostate tissues and 14 prostate tumor tissues were measured by qRT-PCR. Statistical analyses between groups were performed using an ANOVA analysis. Significance was defined as p < 0.05 (*, p < 0.05; **, p < 0.01; ***, p < 0.001).

GAS5 has been reported as a tumor suppressor and was down-regulated in lung[16], gastric[22] and hepatocellular cancer[23]. However, few studies reported the expression levels of GAS5 in prostate cancer. In order to explore the expression of GAS5 in PCa globally, we first analyzed GAS5 expression in four public databases GSE8511, GSE55945[24], GSE38241[25] and TCGA. Our analysis in all the four databases showed that GAS5 was upregulated in PCa tissue samples compared with normal tissue samples (Fig 2A, 2B, 2C and 2D). Meanwhile, TCGA analysis also indicated that the expression level of GAS5 was higher in 419 tumor tissue samples than that in 52 normal tissue samples (Fig 2D).

Besides, the expression of GAS5-007 in four PCa cell lines and the noncancerous prostatic cells WPMY-1 was examined. Our results showed that the expression of GAS5-007 was up-regulated in PCa different cell lines compared to WPMY-1. Interestingly, GAS5-007 also expressed a relatively low level in PC3, DU145, and 22Rv1 cells in comparison to LNCaP cells (Fig 2E). To further validate the expression differentiation between tumor and normal tissue samples, the relative expression level of GAS5-007 was measured by qRT-PCR. Accordingly, the expression of GAS5-007 in 14 tumor tissue samples was higher than that in 11 normal tissue samples (Fig 2F).

### Molecular function analysis of GAS5

**Fig 3:**
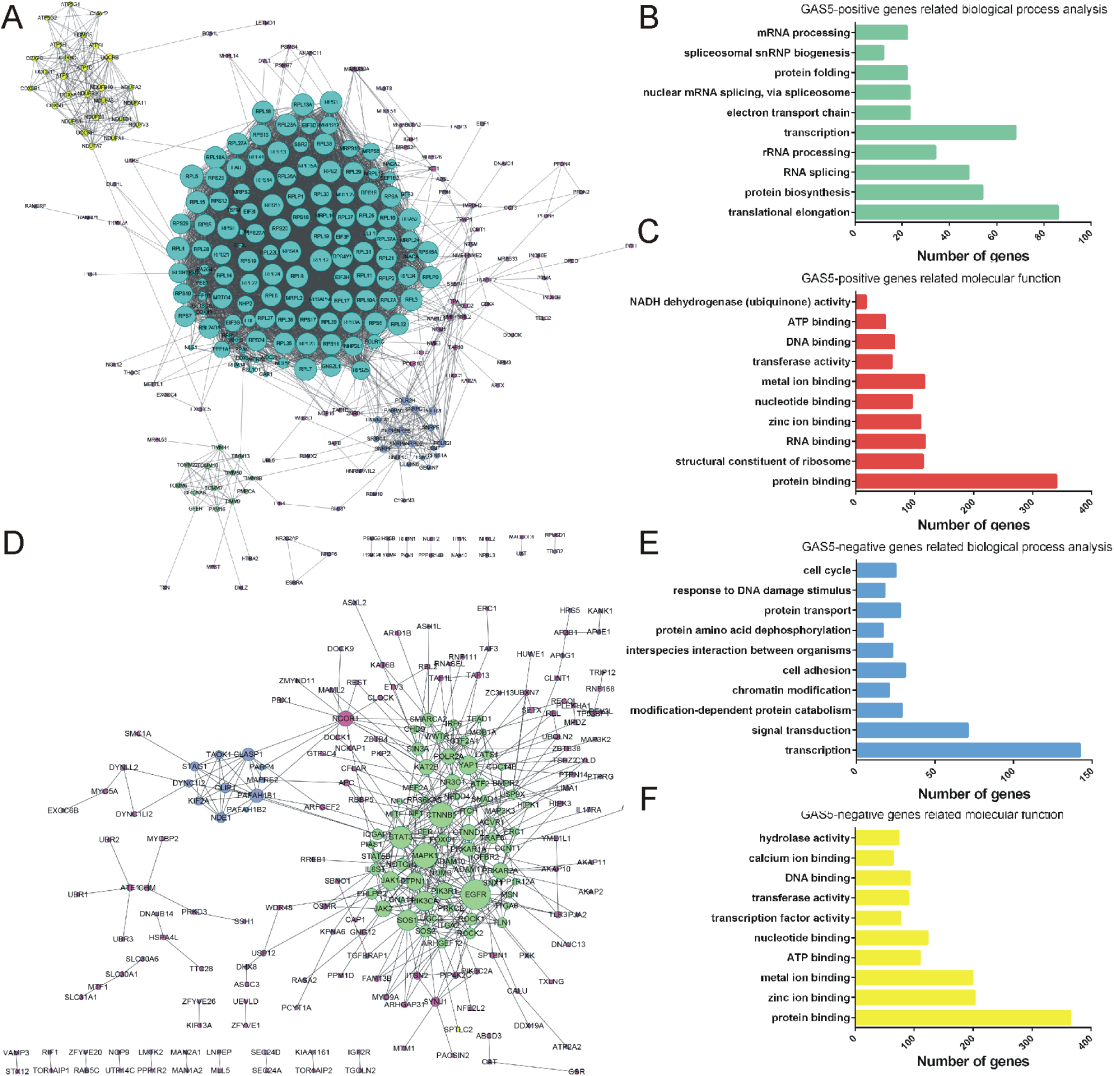
GO biological process and KEGG pathway enrichment analysis of LncRNA GAS5. (A) Gene co-expression networks of GAS5 were constructed according to Pearson correlation coefficients. (B) GO biological process analysis of GAS5-positive genes and GAS5-negative genes (E). (C) KEGG pathway enrichment analysis of GAS5-positive genes and GAS5-negative genes (F) using MAS 3.0. (D) The PPI network.

To predict the functions of GAS5 in PCa, we constructed a gene co-expression network according to Pearson correlation coefficients using TCGA database. Next, the top 1000 positive related genes and the top 1000 negative related genes were classified according to GO term using MAS 3.0 (Fig 3A).

According to our analysis, we found that GAS5 positive related genes were associated with translational elongation, protein biosynthesis, RNA splicing, rRNA processing and transcription (Fig 3B) and GAS5 negative related genes were mainly involved in regulating transcription, signal transduction, modification-dependent protein catabolism, chromatin modification and cell adhesion (Fig 3C). Molecular function analysis suggested that positive related genes of GAS5 were mainly enriched in protein binding, structural constituent of ribosome, RNA binding and zinc ion binding (Fig 3E) and negative related genes of GAS5 were mainly enriched in protein binding, zinc ion binding, metal ion binding and ATP binding (Fig 3F).

To evaluate the interactive relationships among GAS5 co-expressed genes, we mapped the DEGs to STRING, and only experimentally validated interactions with a combined score>0.4 were selected as significance. Then, PPI networks were constructed using the Cytoscape software and showed in Fig 3D.

### GAS5 promoted cell proliferation and inhibited cell apoptosis

**Fig 4:**
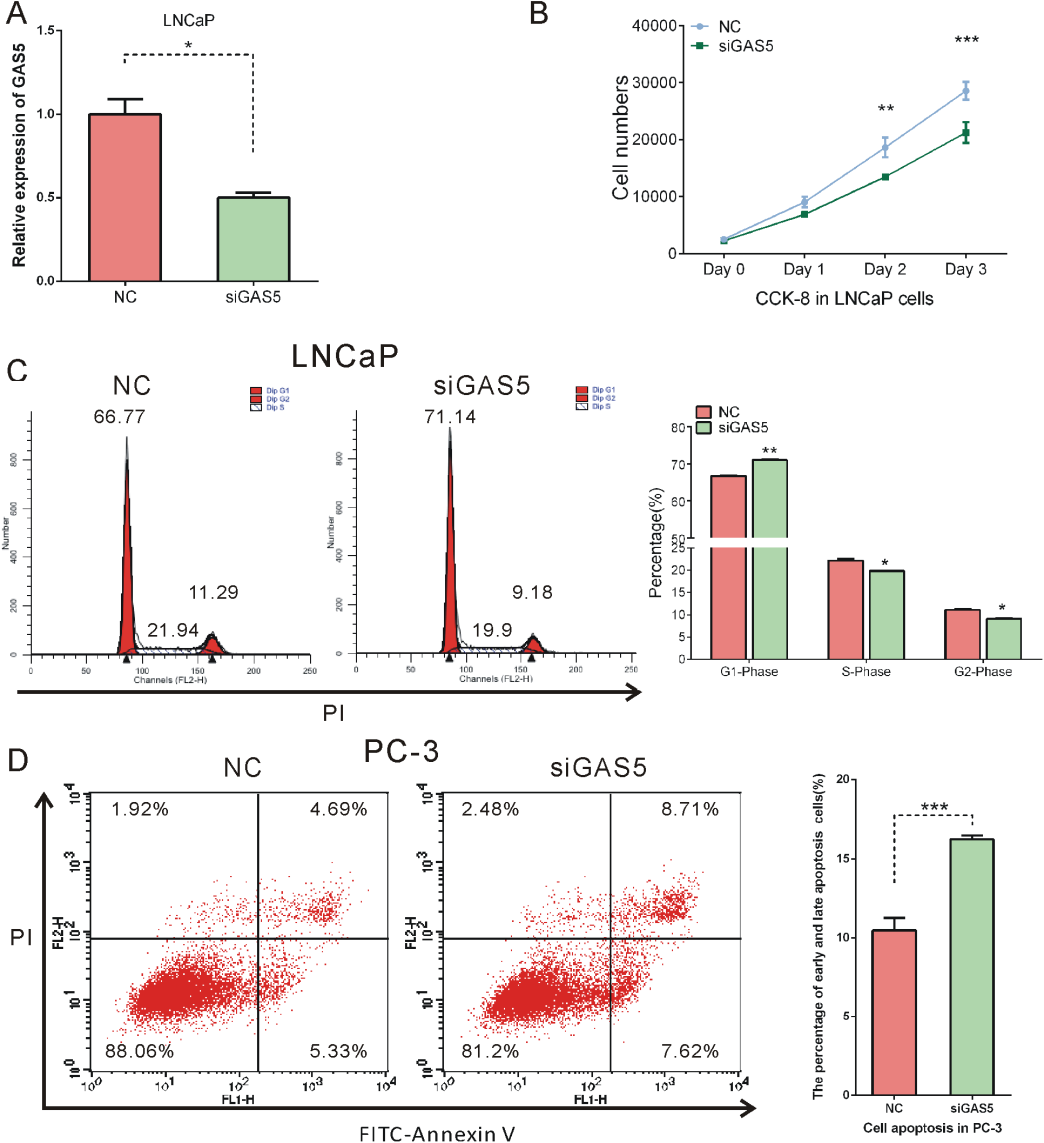
LncRNA GAS5 promoted cell proliferation, cell cycle and inhibited cell apoptosis in PCa cells. (A) The efficiency of siGAS5 was confirmed by qRT-PCR. (B) Knockdown of GAS5 inhibited the proliferation of LNCaP cells. (C) Cell cycle assay was performed in LNCaP cells. Cells were transfected with NC or siGAS5, stained with PI and evaluated with FACScalibur flow cytometer. Knockdown of GAS5 inhibited cell cycle. (D) Cell apoptosis assay was performed in PC-3 cells. Cells were transfected with si-NC or si-GAS5, stained with PI and FITC. GAS5 knockdown increased the percentage of cells in both early apoptosis and late apoptosis. The cell cycle and apoptosis analysis results presented as mean ± SD (n = 3). Significance was defined as p < 0.05 (*p < 0.05; **p < 0.01; ***p < 0.001).

Aiming to evaluate the functions of GAS5-007 in PCa, we transfected siGAS5 to knockdown GAS5 in LNCaP, and PC-3 cells. Interference efficacy was examined by qRT-PCR (S1 Fig) and the result showed that the expression of GAS5-007 was effectively reduced by siGAS5 (Fig 4A).

Then we investigated the role of GAS5-007 in cell proliferation, cell cycle and cell apoptosis in prostate cancer cells. The CCK-8 assay suggested that the cell viability was suppressed in LNCaP cells after knocking down GAS5-007 (Fig 4B). Cell-cycle analysis showed the percentage of LNCaP cells in G1 phase was increased and that in S phase and G2 phase were decreased after knocking down GAS5-007, indicating that cell proliferation was inhibited (Fig 4C). We also analyzed the influence of GAS5-007 on cell apoptosis. As shown in Fig 3D, the percentage of cell in apoptosis was markedly increased after GAS5-007 interference, suggesting that knockdown of GAS5-007 facilitated cell apoptosis. These results revealed a cancer-promoting role of this transcript of GAS5 in PCa.

### Different transcripts of GAS5 regulating different miRNAs in prostate cancer

In this study, we found GAS5-007 acted as an onco-transcript in PCa, which was not consistent with previous study. To explore the molecular mechanisms involved in different transcripts of GAS5 regulating prostate cancer progression, we selected other two transcripts (GAS5-002 and GAS5-001) and constructed lncRNA-miRNA networks based on bioinformatic analysis in this study. GAS5-001 was widely reported as a tumor-suppressive transcript and GAS5-002 was the longest transcript of GAS5.

**Fig 5:**
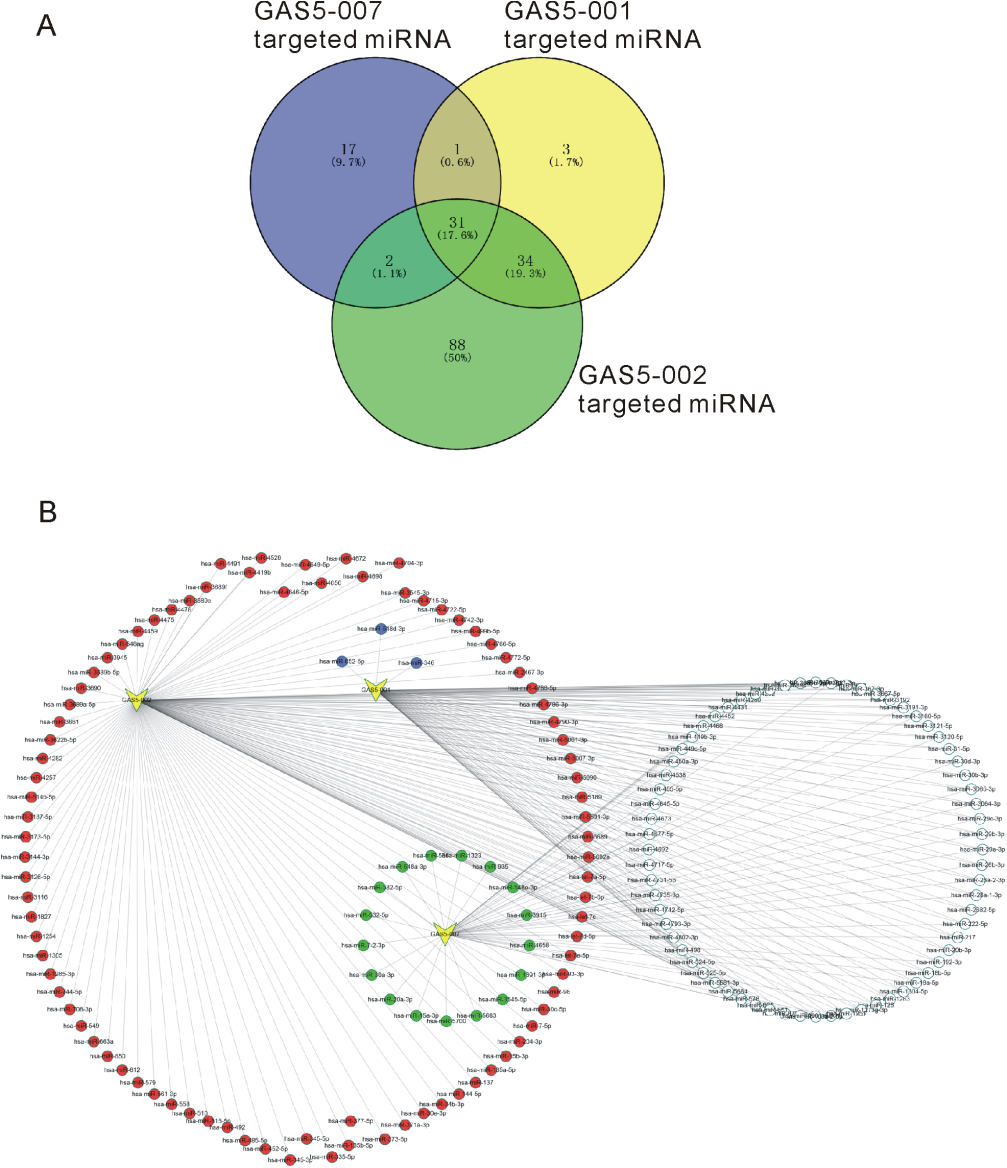
Differences of targeted miRNA of GAS5-001, GAS5-002 and GAS-007. (A) Intersections of GAS5-001, GAS5-002 and GAS5-007 targeted miRNA. (B) Targeted miRNA networks of GAS5-001, GAS5-002 and GAS5-007.

By using RegRNA 2.0, we predicted the potential miRNA regulating GAS5-007, GAS5-002 and GAS5-001. A total of 51 miRNAs, 155 miRNAs and 69 miRNAs were found to regulate GAS5-007, GAS5-002 and GAS5-001, respectively (S1 Table). From the results, we found different transcripts of GAS5 were regulated by different miRNAs in PCa (Fig 5A). Seventeen miRNAs (including hsa-miR-15a-3p, hsa-miR-20a-3p, hsa-miR-30a-3p and hsa-miR-7-2-3p) were found to regulate GAS5-007 expression specially. Three miRNAs (including hsa-miR-346, hsa-miR-652-5p and hsa-miR-548d-3p) were found to regulate GAS5-001 expression specially (Fig 5B).

## Discussion

LncRNA was reported to be involved in the progression of different kinds of cancer[3–5]. In prostate cancer, plenty of LncRNAs, including PCGEM1[9], SOCS2-AS1[10], PlncRNA-1[11], CTBP1-AS[12], DRAIC and PCAT29[13], have been reported to be associated with AR pathway, and thus act as regulators in the development of PCa. In our previous reports, we identified a series of androgen-responsive lncRNAs and found LINC01138 and SUZ12P1 promoted the proliferation of PCa[15]. GAS5-007 was showed to be a dysregulated lncRNA after DHT treatment. Using a qRT-PCR assay, we validated that GAS5-007 was significantly down-regulated after DHT stimulation in both time- and dose-dependent manner and significantly up-regulated after AR knockdown, suggesting GAS5-007 was an androgen-reduced lncRNA.

GAS5 has been brought under the spotlight in recent studies, suggesting it played pivotal roles in different kinds of cancers. According to previous researches, GAS5 has been reported as a tumor suppressor[16–19]. For example, it has been reported to suppress the migration and invasion of hepatocellular carcinoma cells via miR-21[23], inhibit the proliferation of bladder transitional cell carcinoma via regulation of CCL1[26], negatively regulate human glioma cells survival and proliferation[27], enhance G1 cycle arrest by binding to YBX1[28], etc. In prostate cancer, existing studies also suggested GAS5 might act as a tumor suppressor. However, we found GAS5 was significantly upregulated in PCa samples by using public databases. Accordingly, qRT-PCR assay also showed the expression of GAS5-007 in 14 tumor tissue samples was higher than that in 11 normal tissue samples.

To obtain insight into more potential roles of GAS5, we performed co-expression analysis and GO biological process analysis. According to our analysis, we found that GAS5 positive related genes were associated with translational elongation, protein biosynthesis, RNA splicing, protein binding and structural constituent of ribosome. We also found GAS5 negative related genes were mainly involved in regulating transcription, signal transduction, modification-dependent protein catabolism, protein binding, and zinc ion binding.

We explored the functions of GAS5-007 in PCa cells, finding that the proliferation of PCa cells was suppressed and cell apoptosis was facilitated after knocking down GAS5-007. These results revealed a cancer-promoting role of GAS5-007 in PCa, which was not consistent with previous reports. To explore the molecular mechanisms involved in different transcripts of GAS5 regulating prostate cancer progression, we selected other two transcripts (GAS5-002 and GAS5-001) and constructed lncRNA-miRNA networks based on bioinformatic analysis in this study. GAS5-001 was widely reported as a tumor-suppressive transcript and GAS5-002 was the longest transcript of GAS5. From the results, we found different transcripts of GAS5 were regulated by different miRNAs in PCa. Seventeen miRNAs (including hsa-miR-15a-3p, hsa-miR-20a-3p, hsa-miR-30a-3p and hsa-miR-7-2-3p) were found to regulate GAS5-007 expression specially. Of note, hsa-miR-7 had been reported as tumor suppressors in PCa[29].

In conclusion, this study investigated the functions of an androgen-reduced transcription of GAS5. To probe functions of it, we used both prediction through GO, KEGG pathway analysis and functional experiment, including CCK-8 assay, cell cycle and apoptosis analysis. Our results suggested that after knocking down GAS5-007, cell proliferation and cell cycle were inhibited, while cell apoptosis was facilitated. Consequently, we summarized that this transcript of GAS5 promoted proliferation in PCa. We hope that our study will provide a potential new therapeutic and prognostic target for prostate cancer.

## Supporting information

S1 Fig Transfection efficiency of siGAS5. The relative expression level of GAS5 after transfection of siGAS5 in LNCaP cells was tested.
(TIF)

S1 Table GAS5-001, GAS5-002, GAS5-007 binding miRNAs. GAS5-001, GAS5-002, GAS5-007 binding miRNAs were shown in the table respectively.
(XLSX)
